# P03-021 - Characterization of BM-MSC from osteopetrotic mice

**DOI:** 10.1186/1546-0096-11-S1-A219

**Published:** 2013-11-08

**Authors:** F Schena, E Caci, N Lo Iacono, V Marrella, M Gattorno, A Martini, C Sobacchi, A Villa, E Traggiai

**Affiliations:** 1 Second Pediatric Division, Gianna Gaslini Institute, Genova, Italy; 2 Molecular Genetics Laboratory, Gianna Gaslini Institute, Genova, Italy; 3 UOS/IRGB, Milan Unit, CNR, Milano, Italy; 4 Humanitas Clinical and Research Center, Milano, Italy; 5 Novartis Institute for Biomedical Research, Basel, Switzerland

## Introduction

Autosomal Recessive Osteopetrosis (ARO) is a severe bone disease characterized by increased bone density due to impairment in osteoclast bone resorptive function (osteoclast-rich forms) or differentiation (osteoclast-poor forms). The latter form carries mutations in Tnfsf11 gene, which codifies for the receptor activator of NF-κB ligand (RANKL), an essential cytokine expressed in stromal cells. It contributes (with M-CSF) to the differentiation and activation of specialized osteoclasts from monocyte precursors in bone marrow niche. These patients, differently from other forms, do not benefit from HSC transplantation, demonstrating a pathogenetic role of stromal cells in unbalanced bone remodeling.

## Objectives

Aim of the project is to characterize the stromal compartment in a murine model of osteopetrosis, RANKL deficient. In order to verify whether a defect in RANKL protein might cause an impairment in this compartment we generated bone marrow mesenchymal stromal cells (BM-MSC) from knockout mice. We investigated whether nonfunctional RANKL alters physiological features such as morphology and phenotype of MSCs. In order to evaluate if a defect in RANKL affects the physiological functions of MSCs, the clonogenic, proliferative and differentiation potential towards the osteogenic and adipogenic lineages was assayed.

## Methods

MSCs were isolated from bones of wt and ko mice and were cultured in vitro with selective medium. Cell surface markers and proliferation were analyzed through flow citometry and CFSE labeling. Differentiation in vitro was induced with a specific medium for osteoblastic or adipogenic lineage. Alizarin Red and Oil Red staining was performed to confirm the differentiation respectively for OBs and adipocytes.

## Results

7 BM MSC lines from wt and 6 MSCs from ko mice have been generated. We analyzed the immune-phenotype using the following markers: CD45, CD34, CD9, SCA1, CD62L, CD117, CD44 and we observed a comparable phenotype despite a reduction in CD9 expression in *Rankl*-/- MSCs.We tested the clonogenic potential of KO MSCs and we observed a reduced capacity to form CFU, whereas the proliferation of ko MSCs was not different from the one of wt MSCs. Finally we evaluated the differentiation potential in vitro of Rankl -/- MSCs and we found that they are able to differentiate into osteoblasts (OBs) and adipocytes, but the osteoblastogenesis is reduced with respect to wt cells, even if not in significant manner. Indeed we analyzed the expression of osteoblastic markers (RUNX2, OSP, ALP, COL1) and we observed that during the differentiation ko MSCs express OSP and ALP at lower level than wt MSCs.

## Conclusion

These results show that nonfunctional RANKL affects the expression in CD9, influences the intrinsic clonogenic potential of MSCs and the capacity to differentiate to OB suggesting a possible role of RANKL cytokine in stromal cell physiology and a consequent pathogenic role of bone marrow stroma in osteopetrosis.

## Competing interests

None Declared.

